# Role of *Ocimum sanctum* as a Genoprotective Agent on Chlorpyrifos-Induced Genotoxicity

**DOI:** 10.4103/0971-6580.75845

**Published:** 2011

**Authors:** Asha Khanna, Poonam Shukla, Shajiya Tabassum

**Affiliations:** Department of Zoology and Biotechnology, St. Aloysius’ College, Pachpedi, Madhya Pradesh, India; 1 Department of Zoology and Biotechnology, Government Science College, Pachpedi, Madhya Pradesh, India; 2 Department of Zoology and Biotechnology, Mata Gujri Women’s College, Jabalpur, Madhya Pradesh, India

**Keywords:** Chlorpyrifos, chromosomal aberration, comet assay, *Ocimum sanctum*

## Abstract

Protective effect of *Ocimum sanctum* was evaluated on chlorpyrifos-induced genotoxicity in *in vivo* and *in vitro* models. Two different concentrations of pesticide were taken, i.e., 1/5 and 1/15 of LD_50_ of chlorpyrifos for the *in vivo* study. Rats were pre-treated orally with *O. sanctum* extract (OE) at 50 mg/kg b.wt. For the *in vitro* studies, human lymphocyte cultures were exposed to 75 *μ*g/ml chlorpyrifos with and without OE. Structural and numerical (both aneuploidy and euploidy types) chromosomal aberrations (CAs) were scored for the assessment of induced genotoxic effects, while the variation in mitotic index (MI) was considered as a monitor for induced cellular toxicity. The same concentration of the pesticide (75 *μ*g/ml) was taken to study the DNA damage by comet assay. Results showed that lymphocytes treated with the pesticide exhibited increased DNA damage but the increase was statistically insignificant (*P*>0.05). In rats pretreated with OE, a significant (*P*<0.01) increase in MI was observed and there was a significant decrease in the frequency of aberrant cells as compared to the rats treated with chlorpyrifos alone. A significant (*P*<0.05) increase in CA was observed in cultures treated with 75 *μ*g/ml chlorpyrifos as compared to controls, which decreased significantly (*P*<0.05) with OE pretreatment.

## INTRODUCTION

Chlorpyrifos, a non-systemic broad-spectrum organophosphate insecticide, is used for the control of a large number of insect pests of various crops. It is a cholinesterase inhibitor.[1] Since *Ocimum sanctum* leaf extract has time tested healing value in the traditional Indian medicinal system, it was thought that it would be interesting to know if it has a genoprotective effect against aberrations induced by chlorpyrifos in *in vivo* mouse system.

There is a continued interest and need to identify and develop non-toxic genoprotective compounds. An efficient genoprotectant could prove useful in occupational and therapeutic settings where genotoxic chemicals are used or where exposure occurs. *O. sanctum*, commonly called *“Tulsi”* (Family Labiateae), is easily available in the whole of tropical and subtropical India. It is held sacred by Hindus, and various parts of the plant have been traditionally used in *Ayurveda* and *Siddha* systems of medicine for the treatment of diverse hepatic disorders, cold, cough and as an antidote for snakebite.[2] It has also been reported to have anticarcinogenic activity,[3] as well as radioprotective effects.[4] Flavonoids isolated from *O. sanctum* scavenged the free radicals *in vitro* and showed antilipoperoxidant activity *in vivo* at a very low concentration.[5]

It is well known that pesticides are genotoxic to experimental subjects (rat/mice) and have been shown to cause the same effects in human subjects also.[6] The extensive application of pesticides in modern agriculture requires an intensive investigation of the impact of these chemicals on the environment and public health. With the dispersal of hundreds of millions of kilograms each year, these agents must be analyzed for their mutagenic properties.

Therefore, looking at the extensive application of chlorpyrifos and the possible genoprotective role of *O. sanctum*, it was considered worthwhile to undertake this study. Thus, the present study was undertaken to investigate the genoprotective effect of *O. sanctum* extract (OE) on mitotic index (MI) and chromosomal aberration (CA) percentage in bone marrow cells of rats induced with 1/15 and 1/5 LD_50_ of chlorpyrifos. Our goal was also to evaluate the cytogenetic effects of single exposure to chlorpyrifos although pesticide sprayers receive a chronic exposure to this commonly used pesticide.

## MATERIALS AND METHODS

### Comet assay to assess DNA damage

Comet assay or single cell gel electrophoresis assay was used for the evaluation of DNA damage in individual cells.[7] The experimental sample consisted of blood subjected to chlorpyrifos at 75 *μ*g/ml (the concentration is one-fourth of what is sprayed by farmers in the field) for 2 hours at 37°C. Blood sample treated with 40 mM H_2_O_2_ for 10 min at room temperature formed the positive control. The negative control was untreated blood. A small number of cells are immersed in agarose gel, lysed, subjected to an electrophoretic field and then stained with silver stain.[8] The assay was run in triplicate for experimental and control samples. Fifty cells for each sample were scored for DNA damage visually under the light microscope and were classified into six categories[9] as shown below:

Category A: Undamaged cells
Categories B-E: Cells with progressively greater DNA damage
Category F: Apoptotic cells

### *in vitro* lymphocyte culture and *in vivo* studies

#### Preparation of OE

Fresh leaves of *O. sanctum*, collected locally, were air dried, powdered and extracted with 50% ethyl alcohol and 50% distilled water in a soxhlet apparatus by refluxing for 68 hours (at 4 hours/day for 17 days) at 60°C. The extract was evaporated to obtain it in a powder form. For oral administration, the extract was constituted in 0.5 ml distilled water and administered at 50 mg of extract/kg b.wt. to rats, since this dose of OE gave protection against radiation injury.[10] The dose of the insecticide was calculated as 1/15 and 1/5 of the recommended LD_50_(135 mg/kg b.wt.) for rats.

- Group 1 – control, untreated rats
- Group 2 – treated with 1/5 LD_50_ of chlorpyrifos
- Group 3 – pretreated with OE and i.p. injection of 1/5 LD_50_ of chlorpyrifos
- Group 4 – treated with 1/15 LD_50_ of chlorpyrifos
- Group 5 – pretreated with OE and i.p. injection of 1/15 LD_50_ of chlorpyrifos

#### Animals

Experiments were conducted on albino rats weighing approximately 75 g. The rats were acclimatized to the laboratory conditions for 48 hours. They were maintained on standard rat feed and water *ad libitum*. The experimental group for each treatment consisted of six animals. The experiments were conducted according to the recommendations of the institutional ethical committee. The first treatment (1/5 of LD_50_) consisted of an i.p. injection of chlorpyrifos at 27 mg/kg b.wt. The animal was sacrificed 24 hours later and bone marrow chromosome preparations were made as per the standard hypotonic/air drying/Giemsa technique. For the second treatment, rats were fed OE at 50 mg/kg per day for 21 days and thereafter given an i.p. injection of 1/5 LD_50_ of chlorpyrifos. The rats were sacrificed 24 hours later and bone marrow preparations were made in the usual manner. The same experimental schedule was followed for 1/15 LD_50_ of chlorpyrifos. For the controls, the rats were given 0.5 ml distilled water orally for 21 days.

#### Human lymphocyte culture

The assessment of the genoprotective role of OE was also carried out *in vitro* in cultured human lymphocytes. The chromosome preparations were made from peripheral blood cultures following the method of Moorhead *et al*.[11] As a first step, the CA percentage was assessed using 75 *μ*g/ml. In the other samples, *O. sanctum* extract was added at zero hour at 12 *μ*g/ml and chlorpyrifos was added after 48 hours to the culture at 75 *μ*g/ml.

1. Experiment 1: Forty-eight hours after setting up the cultures, the lymphocytes were treated with chlorpyrifos at 75 *μ*g/ml of culture.
2. Experiment 2: Lymphocytes were maintained in culture for 72 hours with OE (added at zero hour).
3. Experiment 3: Forty-eight hours after setting up the cultures, already treated with OE (at zero hour), the lymphocytes were treated with chlorpyrifos at 75 *μ*g/ml of culture, allowing the cells to be in contact with OE for two cell cycles.
4. Untreated (control) 4: Lymphocytes maintained in culture for 72 hours received only distilled water.

CAs were scored under a light microscope at a magnification of 100×. Two hundred metaphase plates were examined per treatment (for *in vivo* studies). Different types of aberrations, such as chromatid breaks, chromosome breaks, fragments and numerical aberrations, were scored to give the total CA percentage for each treatment.

### Statistical analysis

The data were analyzed using student’s *t*-test.

## RESULTS AND DISCUSSION

### Comet assay to assess DNA damage

The assay was run in triplicate for experimental and control samples. Fifty cells for each sample were scored for DNA damage visually under the light microscope and were classified into six categories as shown in Table 1.

**Table 1:** Percentage of cells in different categories of DNA damage

	Cells (%) in different categories of DNA damage					
	A	B	C	D	E	F
Negative control (non-treated)	79.00±2.00	7.00±1.00	5.00±0.33	5.00±0.5	2.00±0.30	2.00±0.50
Treated with chlorpyrifos	63.00±3.50	12.00±1.50	12.00±2.50	5.00±1.00	6.00±0.50	2.00±0.30

### *in vivo* study

#### a) Analysis of mitotic index

For investigation of mitotic index (MI), 3000 cells for each treatment were scored. In the first treatment (1/5 LD_50_), the mean value of MI% in untreated controls was 5.46±0.88, which fell to 1.28±0.52 in animals treated with chlorpyrifos only. The depression caused in MI% by chlorpyrifos treatment was significant (*P*<0.001) as compared to the control values. There was a significant (*P*<0.01) increase in MI% (2.65±0.45) in animals that were given oral OE and 1/5 LD_50_of chlorpyrifos than the MI value of only chlorpyrifos treated rats (1.28±0.52).

In the second treatment (1/15 LD_50_), the mean value of MI% in untreated controls was 5.46±0.88, which fell to 3.88±0.35 in animals treated with chlorpyrifos only. The depression caused in MI% by chlorpyrifos treatment was significant (*P*<0.01) as compared to the control values. There was a significant (*P*<0.05) increase in MI% value (4.30±0.30) in animals that were given oral OE and 1/15 LD_50_of chlorpyrifos than the MI value of only chlorpyrifos treated rats. A significant (*P*<0.05) decrease in MI% value was found in rats treated with 1/5 of LD_50_of chlorpyrifos when compared to those treated with 1/15 LD_50_ of the pesticide [Table 2].

**Table 2:** Mitotic index in bone marrow of different groups of rats

Treatment	MI%
Control	5.46±0.88
Chlor (1/5 LD_50_)	1.28±0.52*
Chlor (1/5 LD_50_) + OE	2.65±0.45Ψ
Chlor (1/15 LD_50_)	3.88±0.35†
Chlor (1/15 LD_50_) + OE	4.30±0.30ф

* *P*<0.001 (comparison between control and chlor 1/5 LD_50_);

Ψ *P*<0.01 (comparison between chlor 1/5 LD_50_ and chlor 1/5 LD_50_ + OE);

† *P*<0.01 (comparison between control and chlor 1/15 LD_50_);

* *P*<0.05 (comparison between chlor 1/15 LD_50_ and chlor 1/15 LD_50_ + OE)

#### b) Analysis of chromosomal aberrations

For the investigation of chromosomal aberrations (CA%), 200 cells for each treatment were scored. In the first treatment (1/5 LD_50_), the mean value of CA for controls was 2.0±0.9 and the value was 27.5±3.0 for animals treated with only chlorpyrifos. Thus, there was a significant increase (*P*<0.001) in the frequency of aberrant cells in bone marrow of rats treated with 1/5 LD_50_ of chlorpyrifos as compared to the control values. However, in the animals pretreated with OE, there was a significant (*P*<0.05) decrease in the frequency of aberrant cells (mean CA% 16.0±1.0) as compared to the chlorpyrifos treated rats.

In the second treatment (1/15 LD_50_), the mean value of CA for controls was 2.0±0.9 and it was 3.5±0.30 for animals treated with only chlorpyrifos. However, there was an increase in the frequency of aberrant cells in bone marrow of rats treated with 1/15 LD_50_ of chlorpyrifos but the increase was not significant. In the animals pretreated with OE, there was a decrease in the frequency of aberrant cells (mean CA% 2.5±0.8) as compared to the chlorpyrifos treated rats [Table 3].

**Table 3:** Frequency of chromosomal aberration in different groups of rats

Group (treatment)	Number of plates	Ctb	Aneu	Hap	Poly	Ct-const	Ctg	PCD	Total CA%
Control	200	–	01	–	–	–	02	01	02.0±0.9
Only 1/5 LD_50_ chlor	200	01	25	09	6	11	02	01	27.5±3.0*
OE and 1/5 LD_50_ chlor	200	01	07	04	01	01	6	12	16.0±1.0**
Only 1/15 LD_50_ chlor	200	–	1	02	1	2	1	–	03.5±0.3
OE and 1/15 LD_50_ chlor	200	–	02	03	–	–	–	–	02.5±0.8

Ctb, chromatid break; Aneu, aneuploidy; Hap, haploidy; Poly, polyploidy, Ct-const, chromatid constriction; PCD, precocious centromere dissociation; Ctg, chromatid gap; CA, chromosomal aberrations;

* *P*<0.001;

** *P*<0.05

### *in vitro* study

A significant increase (*P*<0.05) in CAs was observed in lymphocytes treated with chlorpyrifos and a statistically significant (*P*<0.05) decrease was found in cultures pretreated with OE. MI decreased significantly (*P*<0.05) in cultures treated with chlorpyrifos and a slight increase in MI was found in OE pretreated lymphocytes but the increase was not statistically significant [Table 4].

**Table 4:** Chromosomal aberrations and mitotic index of cultures pretreated with *Ocimum sanctum* extract

Treatment	CA% (mean±SE)	MI% (mean±SE)
Control	1.78±0.17	3.75±0.38
Chlorpyrifos (75 μg/ml)	5.52±0.95*	2.32±0.16Ψ
OE (12 μg/ml) + chlorpyrifos (75 μg/ml)	2.41±0.18**	2.88±0.35

* *P*<0.05 (statistical difference between control and chlorpyrifos);

** *P*<0.05 (statistical difference between chlorpyrifos and OE + chlorpyrifos);

Ψ *P*<0.05 (statistical difference between control and chlorpyrifos); CA, chromosomal aberrations; MI, mitotic index

Table 1shows that in non-treated samples, cells of category A (zero damage) formed 79% of the total cells counted, whereas in the blood samples treated with 75 *μ*g/ml of chlorpyrifos, cells of this category were 63%. The chlorpyrifos treated samples showed 35% cells with DNA damage ranging from categories B-E, whereas in non-treated samples only 19% cells fell in this category (*P*>0.05).

Thus, the study showed that 75 *μ*g/ml of chlorpyrifos caused statistically nonsignificant damage to DNA as determined by comet assay. There seems to be no other reference available on such assessment of genotoxicity of chlorpyrifos. However, similar work has been done on some other pesticides and herbicides which are outlined below.

Assessment of genotoxic effects of chlorpyrifos and acephate by comet assay in mice leukocytes was done by Rahman *et al*.[12] Evaluation of herbicide-induced DNA damage in human lymphocytes by comet assay was done by Ribas *et al*,[13] They found that alachlor, atrazine, maleic hydrazide, paraquat and trifluralin gave positive results for genotoxicity by increasing the comet tail length. Chlorpyrifos-induced DNA damage in rat liver and brain cells was assessed through comet assay by Mehta *et al*.,[14] who classified the DNA damage in various classes from zero to four.

DNA damaging effects of pesticides were measured by comet assay and CAs in Chinese hamster ovary cells by Vigreux *et al*,[15] and they found that chlorothalonil was toxic to CHOK1 cells but carbendazim did not induce DNA strand breaks in comet assay. Occupational exposure of workers employed in pesticide production was found to cause an increase in mean tail length of comet in a study by Paramjit *et al*.[16]

In the classification of pesticides, chlorpyrifos is classified as a “moderately hazardous pesticide” by World Health Organization (WHO).[1] Our results indicate a moderate toxicity of chlorpyrifos at a concentration of 75 *μ*g/ml of blood, which agrees with the WHO data.

This study was also aimed to evaluate the genoprotective effects of *O. sanctum* on chlorpyrifos-induced genotoxicity. The use of pesticides has become a routine mainly in underdeveloped countries, but the genotoxic potential of these substances is not yet well established.[17] Most of the farmers responsible for the application are at risk for cytotoxicity and genotoxicity.

Awa *et al*,[18] detected a positive correlation between the risk of genetic diseases in populations and the level of cytogenetic damage, whereas Au *et al*,[19] hypothesized that CAs were in the background of carcinogenesis and that the determination of their incidence was an important parameter for the effect of various agents on the health status of mammals and man. Thus, the increased frequency of CAs is related to higher risk of development of malignancies.

Maximum number of aneuploidy cells was observed in mice treated with 1/5 of LD_50_ of chlorpyrifos and the number significantly decreased in mice pretreated with OE. There was a total absence of metaphase plates showing precocious centromeric separation in rats pretreated with OE; perhaps the flavonoids and other active components help in polymerization of spindle fibers so that all the cell divisions are in phase.

Radiation and chemical toxins produce biological damage by forming reactive oxygen species like singlet oxygen and superoxides, hydroxyl and hydroperoxy radicals, hydrogen peroxide and organic peroxides.[20]

The genoprotective effect of *O. sanctum* is associated with the presence of its flavonoids, such as orientin and vicenin, which take part in scavenging reactive intermediates that are capable of binding to proteins and DNA.[5] Chlorpyrifos was found to increase the activities of superoxide dismutase, glutathione peroxidase and catalase. Melatonin causes decrease in the above enzymes and an increase in thiobarbituric acid reactive substances. Production of reactive oxygen species could be a cause of DNA damage. *in vitro* and *in vivo* generation of reactive oxygen species, DNA damage and lactate dehydrogenase (LDH) leakage by selected pesticides was studied by Bagchi *et al*.[21] According to them, brain lipid peroxidation and DNA single strand breaks are two indices of oxidative stress and oxidative tissue damage.

Thus, the most likely mechanism of DNA damage and chromosome breakage by chlorpyrifos seems to be through the production of reactive oxygen species, and the present investigation indicates that the pre-treatment of rats with OE at 50 mg/kg per day for 21 days has a significant (*P*<0.001) positive effect in the MI depression caused by chlorpyrifos. *O. sanctum* also had a genoprotective effect on the CA%. It was also found out that OE caused a significant decrease in CA% in *in vitro* lymphocyte cultures. Thus, the genoprotective effect of *O. sanctum* was confirmed both *in vivo* and *in vitro*.
